# An internally validated nomogram for predicting impaired wound healing after calcaneal fracture surgery in patients with type 2 diabetes Mellitus

**DOI:** 10.3389/fsurg.2026.1761198

**Published:** 2026-08-19

**Authors:** Xiaowei Huang, Heng Wang

**Affiliations:** Department of Orthopaedic Surgery, The First Affiliated Hospital of Soochow University, Suzhou, Jiangsu, China

**Keywords:** calcaneal fracture, impaired wound healing, nomogram, risk prediction, type 2 diabetes mellitus

## Abstract

**Background:**

Impaired wound healing remains a major complication following calcaneal fracture surgery in patients with type 2 diabetes mellitus (T2DM), yet reliable tools for individualized risk prediction are lacking.

**Methods:**

Of 553 T2DM patients screened, 360 were eligible and randomly allocated to a training set (*n* = 269 after excluding 1 for incomplete outcome adjudication; 66 outcome events) and an internal validation set (*n* = 90; 22 outcome events, 24.4%). A nomogram was developed using multivariable logistic regression based on predictors selected via LASSO. Model performance was assessed by area under the ROC curve (AUC), calibration (evaluated by the Brier score and the Hosmer-Lemeshow goodness-of-fit test), and decision curve analysis (DCA).

**Results:**

The final model incorporated five predictors: HbA1c, insulin use, diabetic peripheral neuropathy, C-reactive protein, and platelet count. In the training set, the nomogram achieved an apparent AUC of 0.886 (95% CI: 0.846–0.925),with an optimism-corrected AUC of 0.875 (optimism estimate = 0.011). In the internal validation set, the AUC was 0.891 (95% CI: 0.789–0.965). The model showed favorable calibration in the training set [Brier score = 0.113 (95% CI: 0.088–0.140); Hosmer–Lemeshow test, *χ*^2^ = 7.215, df = 8, P = 0.514] and good calibration in the validation set (Brier score = 0.106 (95% CI: 0.067–0.147);calibration slope = 1.27 (95% CI: 0.75–2.89),intercept = 0.44 (95% CI:–0.24–1.85); Hosmer–Lemeshow test, *χ*^2^ = 10.552, df = 8, P = 0.228), and provided higher net benefit than treat-all or treat-none strategies across threshold probabilities from 0.01 to 0.95in both the training and validation sets. At a threshold of 0.4, it identified approximately one-fifth of patients as high-risk, with a high proportion of events among them.

**Conclusion:**

The internally validated nomogram provides a promising tool for risk stratification of postoperative wound complications in T2DM patients with calcaneal fractures. The model is exploratory and limited to internal validation; external validation in multicenter cohorts is required before routine clinical adoption.

## Introduction

Type 2 diabetes mellitus (T2DM) affects over 400 million adults worldwide and substantially elevates the risk of postoperative wound complications across surgical disciplines. In orthopedic trauma, diabetic patients undergoing fracture fixation experience wound healing impairment at rates approximately 2–4 times higher than their non-diabetic counterparts (1, 2), with infection-related morbidity being the principal driver of excess length of stay and readmission. Calcaneal fractures pose a particular challenge: they are among the most common tarsal bone injuries, predominantly affect working-age adults, and almost invariably require operative management via an extended lateral approach—a technique that demands adequate soft-tissue envelope integrity for successful wound closure (3, 4). In the diabetic population, this anatomical and technical vulnerability converges with systemic metabolic derangements—including chronic hyperglycemia-induced microangiopathy, impaired neutrophil function, diminished collagen synthesis, and dysregulated inflammatory responses—to create a uniquely high-risk clinical scenario (5, 6). Reported rates of postoperative wound complications after calcaneal ORIF in diabetic patients range from 16% to 45%, compared with 4%–12% in non-diabetic patients, and these complications frequently progress to deep infection, osteomyelitis, or even amputation when not promptly recognized and managed (4, 7).

Despite refinements in surgical technique, timing optimization, and perioperative protocols, the ability to predict which diabetic patients will develop impaired wound healing after calcaneal fracture surgery remains inadequate. Current clinical practice relies largely on subjective surgeon judgment informed by isolated risk factors such as HbA1c levels or peripheral vascular status, yet these single-variable approaches fail to capture the multidimensional pathophysiology that underlies wound vulnerability in this population (7). Several existing risk-prediction models have been proposed for general orthopedic wound complications or diabetic foot ulcer recurrence; however, none has been specifically developed and validated for postoperative wound healing following calcaneal fracture surgery in T2DM patients. Generic surgical site infection prediction models, such as the National Healthcare Safety Network (NHSN) risk index or the Study on the Efficacy of Nosocomial Infection Control (SENIC) score, were designed for heterogeneous surgical populations and do not incorporate diabetes-specific metabolic variables or procedure-specific factors relevant to calcaneal fracture fixation (8, 9). Furthermore, most existing models report only discrimination metrics (e.g., area under the receiver operating characteristic curve) without rigorous calibration assessment or formal evaluation of clinical utility through decision-curve analysis—key requirements for a prediction tool to be adopted into routine practice (9, 10). Consequently, there remains a critical unmet need for a dedicated, internally validated nomogram that integrates readily available preoperative clinical and laboratory variables to provide individualized, quantified estimates of wound healing risk in this specific high-risk population, thereby enabling targeted preoperative optimization and informed shared decision-making between surgeons and patients.

In the present study, we addressed this knowledge gap by developing and internally validating a nomogram-based prediction model for impaired wound healing following calcaneal ORIF in T2DM patients using a retrospective cohort design with bootstrap correction and split-sample internal validation. Candidate predictors were selected based on biological plausibility and prior evidence, refined through LASSO regularization to minimize overfitting, and incorporated into a user-friendly graphical nomogram suitable for bedside application. Model performance was evaluated comprehensively through discrimination (AUC), calibration (Brier score, Hosmer–Lemeshow test, calibration plot), and clinical utility (decision curve analysis), consistent with the TRIPOD reporting guidelines for transparent prediction model development (9, 10). We hypothesized that a multivariable model incorporating diabetes-specific metabolic, hematologic, and neurologic variables would achieve superior discriminative accuracy and clinical usefulness compared with single-factor assessments, providing clinicians with a practical, evidence-based tool for preoperative risk stratification and individualized perioperative management in this vulnerable population (10).

## Materials and methods

### Study design and population

This retrospective cohort study was conducted at a single tertiary care center (The First Affiliated Hospital of Soochow University). We screened 553 adult patients aged ≥18 years with T2DM who underwent surgery for closed calcaneal fractures between January 2015 and December 2025. Type 2 diabetes was defined by a clinical diagnosis, an admission HbA1c level of ≥6.5%, or current use of hypoglycemic medication.

Included patients had imaging-confirmed Sanders type III or IV closed calcaneal fractures, received open reduction and internal fixation via a standardized extended lateral L-shaped approach within 14 days of injury, and were operated on by the same senior surgical team to minimize inter-surgeon variability (3). All included cases had complete preoperative data. Exclusion criteria included open fractures according to the Gustilo–Anderson classification, prior surgery or chronic pathology affecting the ipsilateral heel, active local or systemic infection at the time of surgery, major organ dysfunction such as end-stage renal disease requiring dialysis or Child–Pugh C liver cirrhosis, recent use of immunosuppressants or systemic corticosteroids, postoperative death within 72 h, follow-up shorter than 30 days without clear documentation of wound outcome, and missing data for core predictor variables (8). To ensure statistical independence and avoid bias from repeated measures, only the first eligible surgical event per patient was retained in the analysis (10).

**Figure 1:**
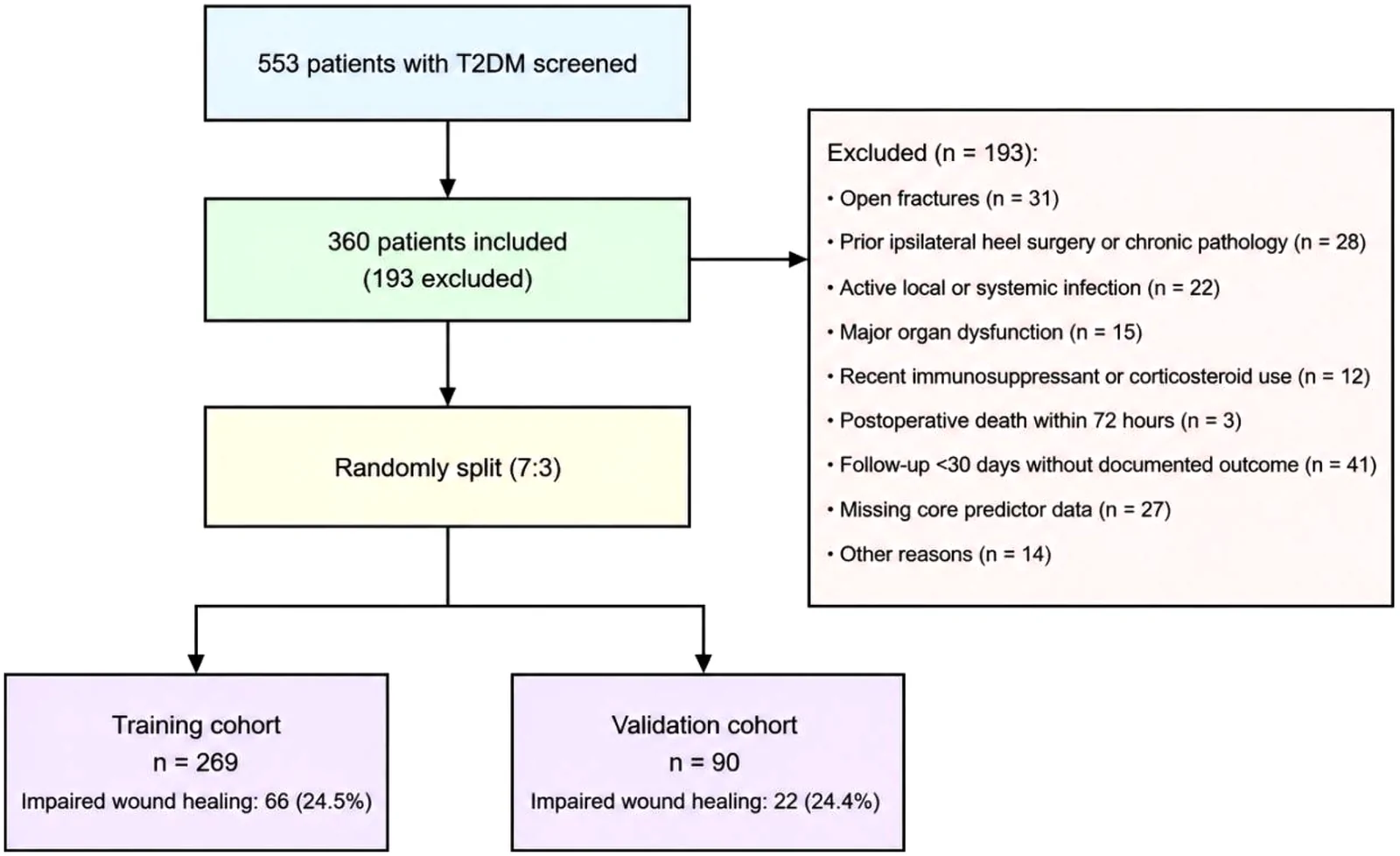
Flowchart of patient selection and study design.

After applying these criteria, 360 patients were included (Figure 1). The 193 exclusions comprised open fractures (*n* = 31), prior ipsilateral heel surgery or chronic pathology (*n* = 28), active local or systemic infection (*n* = 22), major organ dysfunction (*n* = 15), recent immunosuppressant or corticosteroid use (*n* = 12), postoperative death within 72 h (*n* = 3), follow-up shorter than 30 days without documented wound outcome (*n* = 41), missing core predictor data (*n* = 27), and other reasons (*n* = 14). The cohort was randomly divided into a training set (*n* = 270) for nomogram development and an internal validation set (*n* = 90) for performance assessment. One patient in the training set was excluded due to incomplete outcome adjudication, yielding a final analytical sample of 269 patients for model construction, among whom 66 (24.5%) experienced impaired wound healing. The validation set (*n* = 90) was held out entirely and not used in model development; among these 90 patients, 22 (24.4%) experienced impaired wound healing, confirming comparable outcome prevalence between the two sets.

As for soft tissue status assessment, all patients underwent standardized preoperative evaluation of soft tissue condition. The presence and degree of pericalcaneal edema was assessed clinically by the senior surgical team at the time of admission. The timing from injury to surgery was recorded. Tourniquet use was standardized across all procedures, with a maximum tourniquet time of 90 min. Operative duration was documented from incision to closure. The fracture distribution included Sanders type III (*n* = 214, 59.4%) and type IV (*n* = 146, 40.6%) fractures. The median operative duration was 135 min (interquartile range, 108–168 min), and the median tourniquet time was 82 min (interquartile range, 68–95 min). Peripheral vascular disease was assessed in all patients via palpation of dorsalis pedis and posterior tibial pulses and, when clinically indicated, ankle-brachial index measurement; patients with ABI < 0.9 were documented as having peripheral vascular disease (*n* = 12, 3.3%). Prophylactic antibiotics followed a uniform institutional protocol: a single dose of cefazolin 2 g intravenously 30 min before incision, with redosing every 4 h intraoperatively for prolonged procedures. Postoperative wound care comprised sterile dressing changes every 48 h for the first 14 days, with transition to non-sterile wound observation thereafter; no drains were used in any case.

### Data collection and outcome

Clinical, demographic, and laboratory data were systematically extracted from the hospital's electronic medical record (EMR) system. Demographic information and comorbidities were ascertained through admission records and structured patient interviews. Clinical measurements, including body mass index (BMI) and blood pressure, were recorded during the preoperative assessment. Laboratory parameters such as complete blood count, glycemic markers, lipid profile, and renal function indices were obtained from venous blood samples drawn after admission but before surgery. All assays were performed in the hospital's central laboratory using standardized, quality-controlled protocols.

The primary outcome was postoperative impaired wound healing, defined as the occurrence of at least one of the following within 30 days after surgery: delayed epithelialization, wound dehiscence, tissue necrosis, or non-healing ulceration, irrespective of infection status. Wound outcomes were assessed during routine outpatient follow-up visits or, when necessary, through structured telephone interviews. All assessments were documented in the EMR by clinicians who were blinded to the prediction model. The distribution of specific wound complication subtypes in the training set was as follows: delayed epithelialization (*n* = 28, 42.4%), wound dehiscence (*n* = 18, 27.3%), tissue necrosis (*n* = 12, 18.2%), and non-healing ulceration (*n* = 8, 12.1%); 14 cases (21.2%) were associated with concurrent superficial infection (infectious), and 52 cases (78.8%) were non-infectious in origin. A sensitivity analysis comparing the prediction model performance separately in infectious vs. non-infectious wound complications showed consistent discriminative ability (AUC 0.851 (95% CI: 0.768–0.915) and 0.831 (95% CI: 0.773–0.884), respectively). However, the infectious subgroup contained only 14–23 events across datasets, yielding an events-per-variable ratio well below the recommended minimum of 10 for the five-predictor model; therefore, this sensitivity analysis is underpowered and the subgroup-specific AUC estimates should be interpreted with caution. Nonetheless, the consistent direction of discriminative ability across subtypes supports the clinical plausibility of the composite outcome definition.

### Candidate predictors

Based on clinical relevance and existing literature, a prespecified set of 28 candidate predictors was selected to capture factors potentially influencing postoperative wound healing in patients with type 2 diabetes undergoing calcaneal fracture fixation. These included demographic variables (age, sex), diabetes-related characteristics [disease duration, insulin use, and diabetic peripheral neuropathy—diagnosed according to the 2017 ADA/AANEM consensus criteria, requiring the presence of at least two of the following: symptoms of peripheral neuropathy, abnormal neurological examination, and a 10-g Semmes–Weinstein monofilament test result; patients without available monofilament testing data were classified based on symptoms and neurological examination alone, and a sensitivity analysis confirmed that excluding these patients did not materially alter the findings (see Supplementary Material)], and lifestyle factors: smoking status (smoker vs. non-smoker) and alcohol consumption (drinker vs. non-drinker). Additional clinical variables included body mass index, systolic and diastolic blood pressure, and the interval from hospital admission to surgery. A panel of laboratory markers was also incorporated to reflect systemic inflammation (white blood cell count, C-reactive protein), hematologic status (hemoglobin, platelet count), glycemic control (HbA1c, fasting glucose, C-peptide), lipid profile (total cholesterol, triglycerides, HDL, LDL), renal function (creatinine, blood urea nitrogen, estimated glomerular filtration rate, uric acid), and nutritional status (serum albumin). All continuous variables were assessed for normality, and those with significant skewness were log-transformed prior to modeling.

### Predictor selection, model development, and internal validation

To minimize overfitting and derive a parsimonious set of predictors, we employed least absolute shrinkage and selection operator (Lasso) logistic regression with 10-fold cross-validation. The optimal regularization parameter (*λ*) was selected using the one-standard-error rule, which identifies the most interpretable model whose cross-validated performance remains within one standard error of the minimum. Only variables with non-zero coefficients at the selected *λ* were retained and incorporated into the final multivariable logistic regression model. Missing data were minimal (<2% for any individual variable) and were handled using complete-case analysis, as all included patients were required to have complete preoperative data per the inclusion criteria. To assess whether continuous predictors required non-linear modeling, restricted cubic spline terms were evaluated for each continuous predictor in univariable logistic regression models; no statistically significant non-linear effects were detected (all *P* for non-linearity > 0.05), supporting the use of linear terms throughout. Multicollinearity among candidate predictors was examined using variance inflation factors (VIF); all VIF values were below 5, indicating the absence of problematic collinearity. Bootstrap resampling (1,000 iterations) was used to estimate optimism and correct performance metrics; the optimism estimate was defined as the mean difference between apparent and bootstrap performance, and the corrected estimate was calculated as the apparent estimate minus the optimism estimate.

### Sample size considerations

A total of 66 outcome events among 269 patients in the training set yields an events-per-variable (EPV) ratio of 13.2 for the final five-predictor model, exceeding the commonly recommended minimum of 10 EPV (11). Although the initial pool of 28 candidate predictors corresponded to an EPV of approximately 2.4, several safeguards were implemented to mitigate overfitting risk. First, LASSO regularization with 10-fold cross-validation and the one-SE rule inherently penalizes model complexity and selects only predictors that demonstrate robust predictive signal. Second, internal validation was performed using bootstrap resampling (1,000 iterations) with optimism correction to quantify and adjust for any residual overfitting. Third, a hold-out validation set (*n* = 90) completely independent of the modeling process was used to assess generalizability. These measures collectively ensure that the reported performance metrics are not inflated by overfitting despite the modest absolute event count.

A clinical nomogram was subsequently developed based on this model to enable individualized prediction of the probability of postoperative impaired wound healing. Model performance was rigorously assessed through comprehensive internal validation. Discriminative ability was quantified by the area under the receiver operating characteristic curve (AUC) with 95% confidence intervals. Calibration—reflecting the agreement between predicted risks and observed outcomes—was evaluated using a calibration plot, the Brier score, and the Hosmer–Lemeshow goodness-of-fit test, with optimism corrected via 1,000 bootstrap resamples. Clinical utility was further examined using decision curve analysis (DCA), which estimates the net benefit of the model across a range of clinically relevant risk thresholds. Complementarily, a cost-benefit analysis was conducted to illustrate the trade-off between the number of patients classified as high-risk and the actual incidence of impaired wound healing at varying probability thresholds.

### Statistical software

All analyses were performed using R software (version 4.x; R Foundation for Statistical Computing). Key packages included glmnet (for Lasso regression), rms (for nomogram and validation), and dca (for decision curve analysis). A two-sided *P* < 0.05 was considered statistically significant.

## Results

Among the 269 patients undergoing surgery, 66 (24.5%) experienced postoperative complications related to impaired wound healing, including surgical site infection, delayed closure, or dehiscence. Patients with impaired wound healing were significantly more likely to be receiving insulin therapy (*P* < 0.001), reflecting more advanced or poorly controlled diabetes. Consistent with this, glycemic control was notably worse in this group, as indicated by higher HbA1c levels (*P* < 0.001). Systemic inflammation was also markedly elevated, with significantly higher C-reactive protein concentrations (*P* = 0.035). Additionally, patients with impaired wound healing had lower platelet counts, suggesting thrombocytopenia (*P* < 0.001). In contrast, no significant differences were observed in age, sex, smoking status, body mass index, or most lipid profile components, and total cholesterol which showed no significant difference (*P* = 0.376). These findings suggest that metabolic and inflammatory dysregulation, rather than demographic or lifestyle factors, may play a central role in the pathogenesis of poor wound outcomes in this diabetic cohort.

Of the 269 patients included in the analysis, 66 (24.5%) developed postoperative impaired wound healing, defined as delayed epithelialization, wound dehiscence, tissue necrosis, or non-healing ulcers within 30 days after surgery, regardless of infection status. As summarized in Tables 1, 2, patients with impaired wound healing exhibited distinct metabolic and inflammatory profiles compared to those with uncomplicated healing. Insulin use was significantly more prevalent in the impaired healing group (*P* < 0.001), suggesting a link between advanced diabetes severity and poor tissue repair. This association was further supported by significantly higher HbA1c levels (*P* < 0.001), indicating that chronic hyperglycemia may impair healing mechanisms independently of acute glucose fluctuations, as neither fasting glucose (*P* = 0.013) nor C-peptide (*P* > 0.84) differed between groups. Systemic inflammation was also markedly elevated, with significantly higher C-reactive protein levels (*P* = 0.035). Concurrently, platelet counts were significantly lower in patients with wound complications (*P* < 0.001), potentially reflecting consumption during dysregulated tissue repair or subclinical inflammation. Additionally, total cholesterol did not differ significantly in this group (*P* = 0.376), whereas other lipid parameters showed no significant differences. In contrast, demographic and lifestyle factors—including age, sex, smoking status, alcohol use, body mass index, and blood pressure—were well balanced between groups (age *P* = 0.035; all other *P* > 0.05), reinforcing that metabolic and inflammatory dysregulation, rather than baseline demographics, may be key drivers of wound complications in this diabetic surgical population.

**Table 1:** Baseline demographic and clinical characteristics by impaired wound healing Status .

Variable	Uncomplicated healing (*N* = 203)	Impaired wound healing (*N* = 66)	*P* value
Demographics			
Male sex, *n* (%)	123 (60.6)	39 (59.1)	0.943
Age, years	60.1 ± 13.0	56.1 ± 14.3	0.035
Diabetes-related factors			
Duration of diabetes mellitus, years	8.86 ± 7.17	7.72 ± 7.83	0.297
Insulin use, *n* (%)	12 (5.9)	16 (24.2)	<0.001
Diabetic peripheral neuropathy, *n* (%)	55 (27.1)	36 (54.5)	<0.001
Lifestyle and comorbidities			
Current smoker, *n* (%)	60 (29.6)	21 (31.8)	0.847
Alcohol consumption, *n* (%)	58 (28.6)	13 (19.7)	0.208
Clinical measurements			
Systolic blood pressure, mm Hg	136 ± 19.3	136 ± 18.7	0.904
Diastolic blood pressure, mm Hg	78.0 ± 11.5	77.5 ± 10.1	0.759
Body mass index, kg/m^2^	26.0 ± 6.2	24.7 ± 6.6	0.172
Preoperative hospital stay, days	14.1 ± 15.7	16.2 ± 15.1	0.341
The timing from injury to surgery	7.5 ± 5.9	8.4 ± 5.6	0.303

**Table 2:** Laboratory findings by postoperative impaired wound healing status.

Variable	Uncomplicated healing (*N* = 203)	Impaired wound healing (*N* = 66)	*P* value
Inflammatory markers			
White blood cell count, ×10^9^/L	8.32 ± 2.85	8.29 ± 3.12	0.942
C-reactive protein, mg/L	9.4 ± 8.2	11.9 ± 8.5	0.035
Hematologic parameters			
Hemoglobin, g/L	129 ± 17.8	131 ± 15.2	0.509
Platelet count, ×10^9^/L	222.6 ± 59.4	173.3 ± 54.2	<0.001
Glycemic control			
HbA1c, %	9.1 ± 2.3	10.8 ± 2.3	<0.001
Fasting blood glucose, mmol/L	9.2 ± 4.4	10.8 ± 5.0	0.013
Fasting C-peptide, ng/mL	1.88 ± 1.51	1.85 ± 1.19	0.856
2-h postprandial C-peptide, ng/mL	4.46 ± 3.18	4.37 ± 3.10	0.847
Lipid profile			
Total cholesterol, mmol/L	4.8 ± 1.4	4.6 ± 1.4	0.374
Triglycerides, mmol/L	2.03 ± 2.40	3.07 ± 4.40	0.070
HDL cholesterol, mmol/L	1.18 ± 0.54	1.14 ± 0.36	0.435
LDL cholesterol, mmol/L	3.73 ± 1.20	2.88 ± 1.10	0.123
Renal function			
Serum creatinine, μmol/L	69.8 ± 33.9	64.3 ± 20.9	0.116
Blood urea nitrogen, mmol/L	10.8 ± 3.50	5.73 ± 2.02	0.081
eGFR, mL/min/1.73m^2^	108 ± 36.0	116 ± 43.4	0.179
Uric acid, μmol/L	331 ± 107	319 ± 90.5	0.390
Albumin, g/L	44.5 ± 10.3	43.5 ± 4.12	0.255

### Identification of key predictors for impaired wound healing using Lasso logistic regression

**Figure 2:**
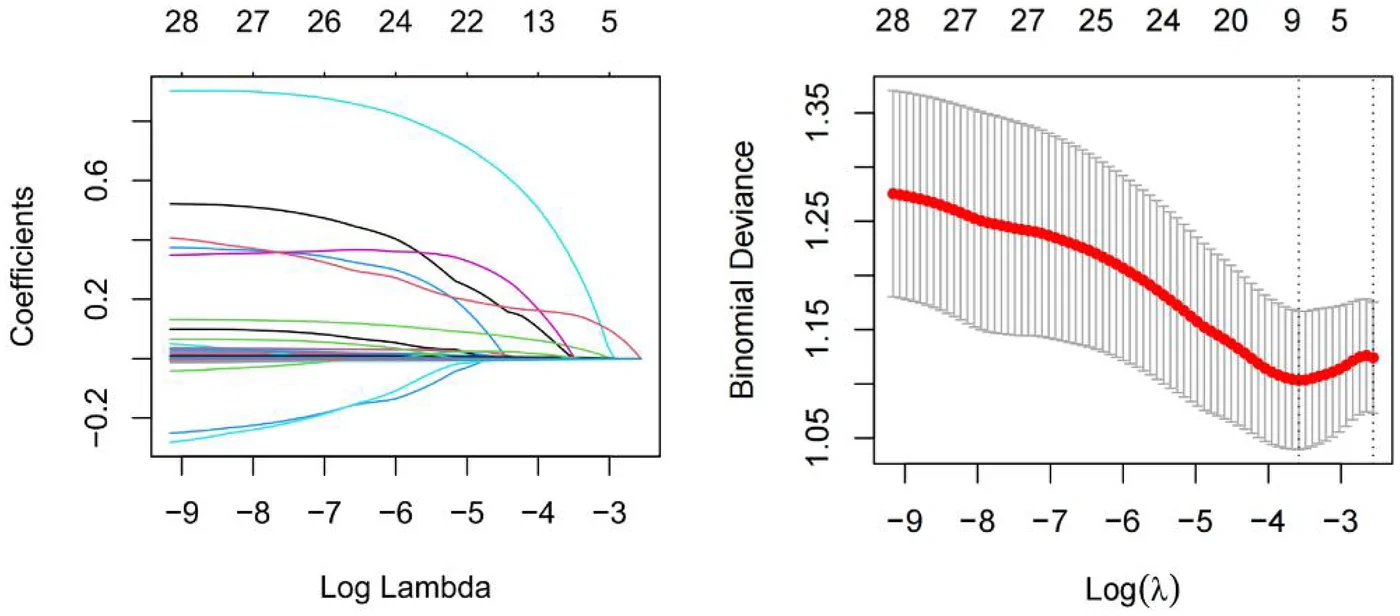
Lasso coefficient paths (left) and cross-validated binomial deviance (right) for variable selection in the prediction model.

Lasso logistic regression was performed to identify key predictors of postoperative impaired wound healing among 269 diabetic patients. The coefficient paths showed that as the regularization parameter decreased, more variables entered the model with non-zero coefficients, as illustrated in Figure 2. At higher values of the regularization parameter, for example at negative three point five, only a small subset of variables retained non-zero coefficients, indicating strong variable selection. The cross-validation plot of binomial deviance displayed a clear minimum at a regularization parameter value of approximately negative four point zero, corresponding to the model with the best fit. However, to balance model simplicity and generalizability, we selected the regularization parameter value located one standard error above this minimum, which was approximately negative three point five. This choice yielded a parsimonious model containing five variables with non-zero coefficients: platelet count, HbA1c, insulin administration, diabetic peripheral neuropathy, and C-reactive protein. These five variables were included in the final multivariable logistic regression model (Table 3).

**Table 3:** Multivariable logistic regression analysis of predictors for postoperative impaired wound healing.

Variable	*β*	OR	95% CI	*P* value
HbA1c (%)	0.478	1.613	1.350–1.926	<0.001
Insulin use	2.465	11.765	3.975–34.819	<0.001
Diabetic peripheral neuropathy	1.493	4.452	2.096–9.459	<0.001
CRP (mg/L)	0.055	1.056	1.013–1.101	0.011
Platelet count (×10^9^/L)	−0.023	0.978	0.970–0.985	<0.001
*Model summary (LLR test)*	0.372	*Pseudo R*^2^ *=* *0.372*		*<0*.*001*

In the final multivariable model, all five predictors demonstrated statistically significant independent associations with impaired wound healing (Table 3). HbA1c emerged as the strongest predictor (OR: 1.613 per 1% increase, 95% CI: 1.350–1.926, *P* < 0.001), indicating that each 1% elevation in glycated hemoglobin corresponded to a 61.3% increase in the odds of impaired wound healing. Insulin use was associated with a more than 11-fold increase in odds (OR: 11.765, 95% CI: 3.975–34.819, *P* < 0.001), reflecting its role as a marker of advanced disease severity. Diabetic peripheral neuropathy independently elevated risk by more than threefold (OR: 4.452, 95% CI: 2.096–9.459, *P* < 0.001), consistent with its established contribution to wound vulnerability through impaired protective sensation and autonomic dysfunction. Elevated C-reactive protein conferred incremental risk per unit increase (OR: 1.056 per 1 mg/L, 95% CI: 1.013–1.101, *P* = 0.011), underscoring the role of systemic inflammation. Lower platelet count was inversely associated with impaired healing (OR: 0.978 per 10^9^/L increment, 95% CI: 0.970–0.985, *P* < 0.001), possibly reflecting platelet consumption during dysregulated tissue repair or subclinical coagulopathy.

### Clinical risk prediction model for postoperative wound healing complications

**Figure 3:**
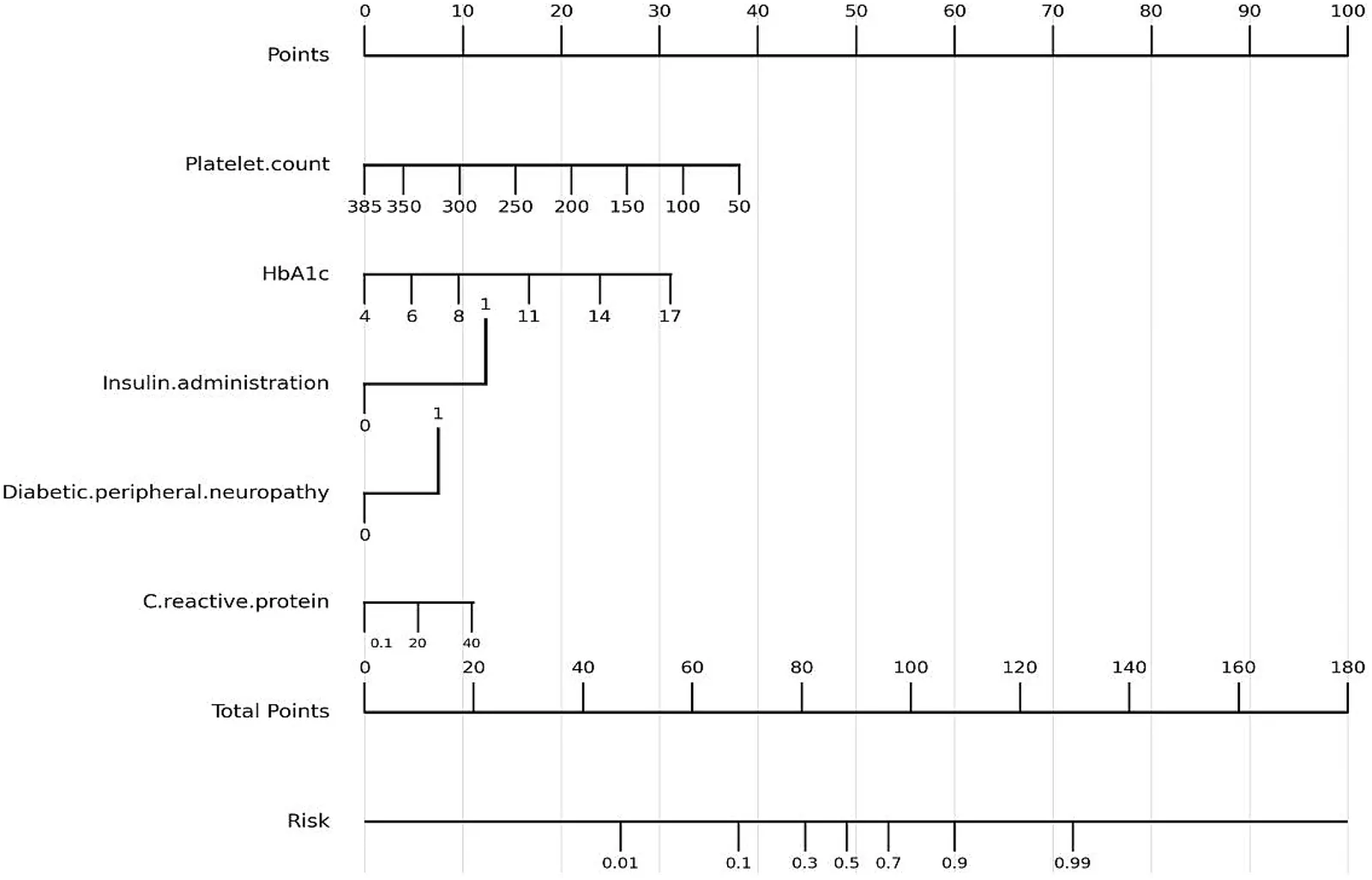
Nomogram for predicting the risk of postoperative impaired wound healing in diabetic patients undergoing calcaneal fracture surgery.

Each predictor was assigned a point value proportional to its contribution to the model, with higher scores indicating greater risk. The total points were summed to generate a composite score, which was then mapped to an estimated probability of impaired wound healing from approximately 0.01 to 0.95. This quantitative tool provides a clinically applicable framework for individualized risk assessment in the perioperative setting (Figure 3).

### Validation of the nomogram's discriminative ability, calibration, and decision-making value

**Figure 4:**
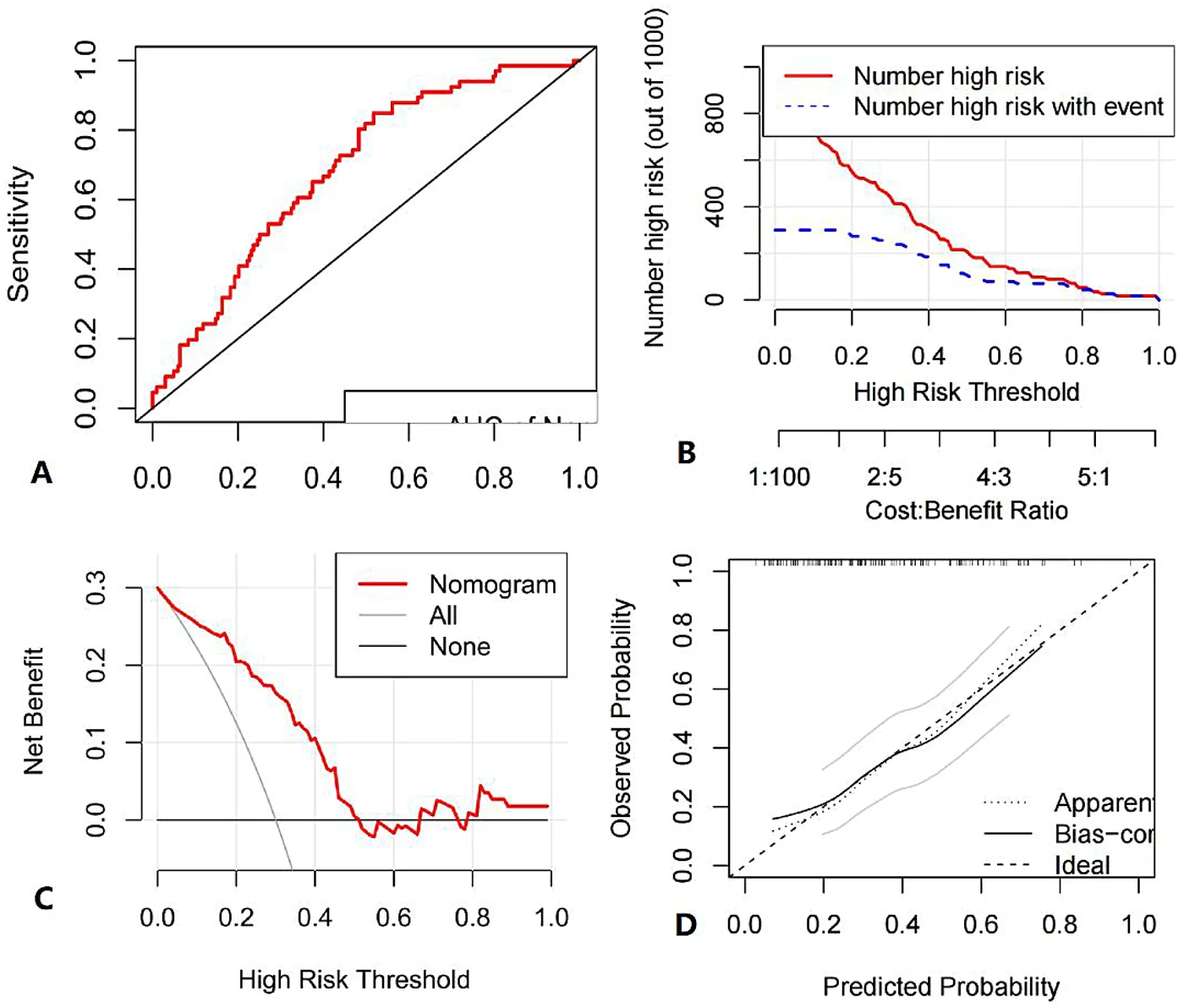
Performance of the nomogram for predicting postoperative impaired wound healing. **(A)** ROC curve in the training set: apparent AUC = 0.886 (95% CI: 0.846–0.925); optimism-corrected AUC = 0.875 (optimism = 0.011). **(B)** Clinical impact curve: number of high-risk patients (red) and those with actual events (blue dashed) per 1000, across threshold probabilities. **(C)** Decision curve analysis: net benefit of the nomogram (red) exceeds “treat all” (gray) and “treat none” (black) for threshold probabilities from 0.01 to 0.95. **(D)** Calibration plot: apparent (dotted, 95% CI bands) and bootstrap-corrected (solid black) curves vs. ideal (gray dashed). Good agreement between predicted and observed probabilities.

In the training set, the predictive performance of the nomogram for postoperative impaired wound healing was rigorously evaluated using discrimination, calibration, and clinical utility metrics (Figure 4). The model demonstrated acceptable discriminative ability, with an apparent area under the receiver operating characteristic curve of 0.886 (95% CI: 0.846–0.925). Bootstrap correction (1,000 resamples) produced an optimism estimate of 0.011 and an optimism-corrected AUC of 0.875. Calibration analysis revealed close agreement between predicted and observed probabilities across the risk spectrum, with the bias-corrected curve closely aligning with the ideal diagonal, indicating minimal over- or under-prediction (Brier score = 0.113; calibration slope = 1.08 (95% CI: 0.79–1.30), intercept = 0.06 (95% CI: –0.41 to 0.46); Hosmer–Lemeshow test, *χ*^2^ = 7.215, df = 8, *P* = 0.514, indicating no significant lack of fit). Decision curve analysis showed that the nomogram provided higher net benefit than treat-all or treat-none strategies across threshold probabilities from 0.01 to 0.95, with the greatest net benefit at low thresholds (<0.3), supporting its potential usefulness for individualized decision-making. A cost-benefit analysis further confirmed a favorable trade-off between intervention intensity and outcome yield, with stable event enrichment among patients classified as high risk across clinically relevant thresholds (Table 4).

**Table 4:** Risk-based clinical management algorithm for patients at risk of impaired wound healing after calcaneal fracture surgery.

Risk category (predicted probability)	Preoperative management	Postoperative monitoring
Low risk (<20%)	Optimize glycemic control if HbA1c elevated (>7.0%)	Routine wound inspection at each postoperative visit
Moderate risk (20–40%)	Target HbA1c < 7.5% before surgery; optimize insulin regimen if indicated Address elevated CRP (infection screen; consider anti-inflammatory strategy); if on insulin, verify dosing	Assess DPN severity; implement foot offloading precautions; more frequent wound inspection (weekly)
High risk (≥40%)	Multidisciplinary optimization (endocrinology + wound care consultation); consider delaying surgery until HbA1c < 8% and CRP trending down If platelet count low: hematology evaluation; DPN: aggressive offloading protocol	If platelet count low: hematology evaluation; DPN: aggressive offloading; daily inpatient wound inspection

### Assessment of model performance through internal validation

**Figure 5:**
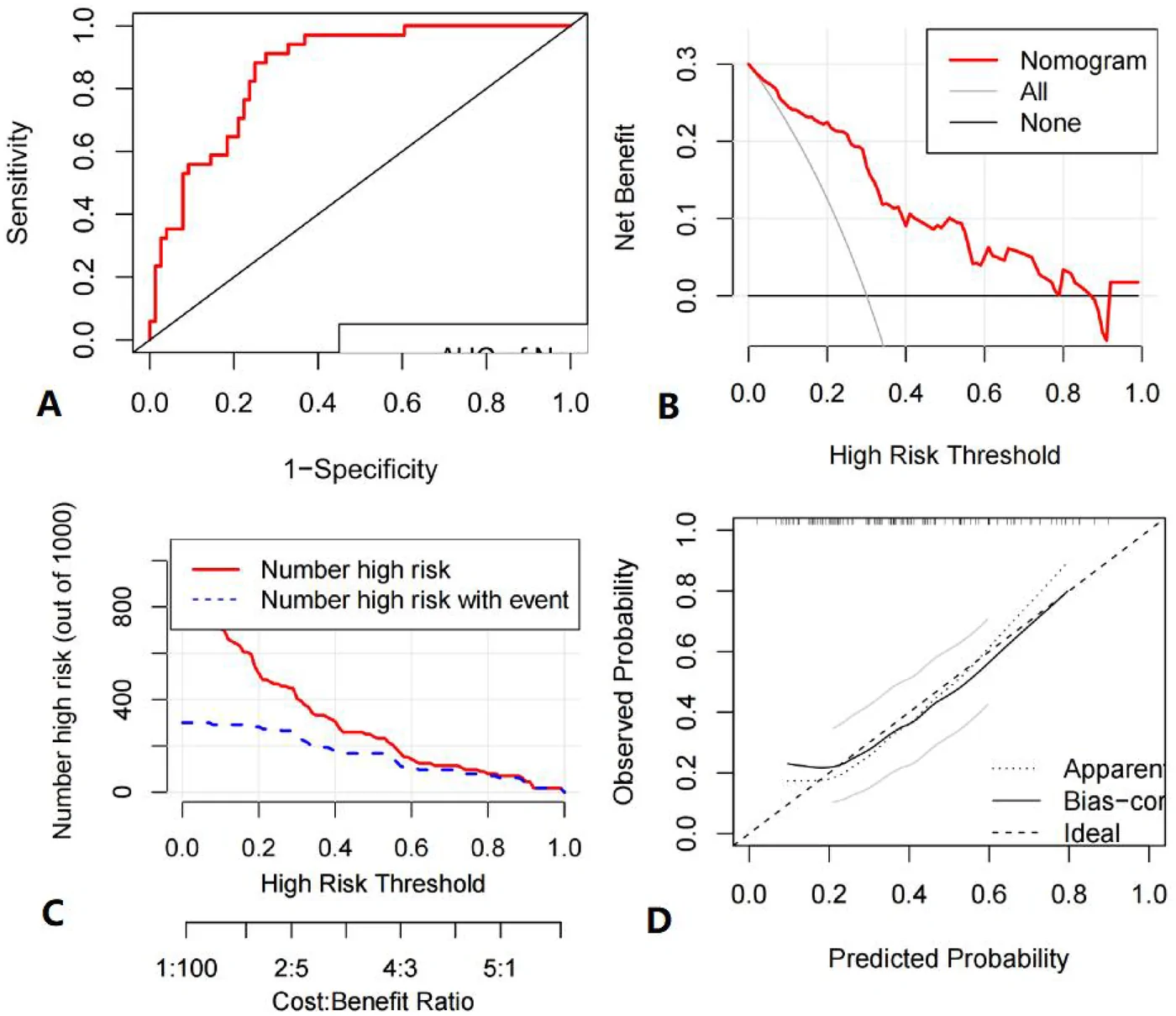
Decision curve analysis of the nomogram for predicting impaired wound healing. **(A)** ROC curve (AUC = 0.891, 95% CI: 0.789–0.965); **(B)** net benefit of the nomogram vs. “treat all” and “treat none” across threshold probabilities from 0.01 to 0.95; **(C)** clinical impact curve (high-risk patients per 1000, with events); **(D)** calibration plot (observed vs. predicted probability). The nomogram demonstrates good discrimination, clinical utility, and satisfactory calibration in the internal validation cohort.

The internal validity of the nomogram was evaluated using discrimination, calibration, and clinical utility metrics based on validation datasets. The model exhibited acceptable discriminative performance in the internal validation set, with an area under the receiver operating characteristic curve of 0.891 (95% CI: 0.789–0.965). Calibration analysis demonstrated acceptable agreement between predicted and observed probabilities (Brier score = 0.106, 95% CI: 0.067–0.147; calibration slope = 1.27 (95% CI: 0.75–2.89), intercept = 0.44 (95% CI:–0.24–1.85); Hosmer–Lemeshow test, *χ*^2^ = 10.552, df = 8, *P* = 0.228). Decision curve analysis confirmed higher net benefit compared with treat-all or treat-none strategies across threshold probabilities from 0.01 to 0.95. Cost-benefit analysis further showed that increasing the risk threshold reduced the number of patients classified as high risk while maintaining a stable event rate among this group, reflecting an efficient trade-off between resource allocation and clinical outcome, as illustrated in Figure 5.

## Discussion

In this cohort of patients with type 2 diabetes undergoing surgical fixation for displaced intra-articular calcaneal fractures, postoperative impaired wound healing emerged as a common and clinically significant complication. Notably, the risk was not attributable to conventional demographic or lifestyle factors such as age, sex, smoking, or body mass index, but was strongly driven by underlying metabolic and inflammatory dysregulation. Patients who developed wound complications were more likely to require insulin therapy and exhibited evidence of chronic hyperglycemia—distinct from acute glycemic fluctuations—highlighting the detrimental role of prolonged glucose toxicity in impairing tissue repair. Concurrently, an altered systemic milieu characterized by elevated inflammatory markers and reduced platelet levels further illustrates the complex interplay between advanced diabetes pathophysiology and compromised wound healing. Collectively, these findings indicate that the biological burden of long-standing diabetes, rather than isolated clinical characteristics, underlies susceptibility to soft tissue complications after complex foot surgery. This supports a paradigm shift in preoperative risk stratification toward objective, readily available biomarkers such as HbA1c and C-reactive protein, which may more accurately identify high-risk individuals. Accordingly, targeted preoperative optimization of glycemic control and modulation of systemic inflammation represent actionable strategies to mitigate complications and improve surgical outcomes in this vulnerable population.

The five predictors retained in the final model—HbA1c, insulin use, diabetic peripheral neuropathy, C-reactive protein (CRP), and platelet count—collectively capture the multifactorial pathophysiology that predisposes patients with type 2 diabetes to impaired wound healing following calcaneal fracture surgery. Elevated HbA1c reflects chronic hyperglycemia, which disrupts multiple phases of tissue repair through mechanisms including immune dysfunction, reduced collagen synthesis, and impaired angiogenesis, largely mediated by the accumulation of advanced glycation end-products and microvascular compromise (12). Insulin use, while not directly causative, serves as a robust clinical surrogate for disease severity—typically indicating longer duration, *β*-cell failure, and greater metabolic instability—and thus identifies individuals at inherently elevated risk (5). Diabetic peripheral neuropathy further exacerbates vulnerability by impairing both sensory and autonomic protective functions: loss of protective sensation increases mechanical trauma risk, while autonomic dysfunction contributes to xerosis, skin fragility, and blunted neurogenic vasodilation, all of which compromise local tissue resilience (13). Systemic inflammation, indexed by elevated CRP, signifies a chronic low-grade pro-inflammatory state driven by adipose tissue dysregulation and oxidative stress; this milieu prolongs the inflammatory phase of healing and suppresses fibroblast and keratinocyte activity, thereby creating an unfavorable microenvironment for regeneration—even in the absence of clinical infection (14). Finally, reduced platelet counts may reflect subclinical endothelial activation, platelet consumption, or diabetes-associated megakaryopoietic dysfunction. Given that platelets are essential reservoirs of growth factors—including platelet-derived growth factor (PDGF), vascular endothelial growth factor (VEGF), and transforming growth factor-beta (TGF-*β*)—thrombocytopenia likely limits the local bioavailability of these mediators, directly impeding re-epithelialization and neovascularization (11). Together, these interrelated factors delineate a “metainflammatory” phenotype—characterized by the convergence of metabolic, neural, vascular, and inflammatory disturbances—that underlies poor surgical wound outcomes in this high-risk population (12).

Our internally validated nomogram demonstrated satisfactory predictive performance for postoperative impaired wound healing in diabetic patients undergoing calcaneal fracture surgery. It achieved an apparent AUC of 0.886 (95% CI: 0.846–0.925) in the training set, with an optimism-corrected AUC of 0.875 (optimism estimate = 0.011), and an AUC of 0.891 (95% CI: 0.789–0.965) in the internal validation set, reflecting acceptable discriminative ability. The model showed good calibration after bias correction, as evidenced by a low Brier score (0.113 in the training set and 0.106 in the validation set) and non-significant Hosmer–Lemeshow tests (*P* = 0.514 and *P* = 0.228, respectively), indicating that predicted risks aligned reasonably well with observed outcomes (15). Internal validation using bootstrapping confirmed modest overfitting (16). Decision curve analysis suggested that the model provided higher net benefit than default strategies across threshold probabilities from 0.01 to 0.95 in both the training and validation sets (11). At a threshold of 0.4, the nomogram identified approximately one-fifth of patients as high-risk, with a high proportion of events in this group, which may support targeted perioperative attention (17). These results are promising; however, given the single-center, retrospective, internally validated design and the pseudo-R^2^ of 0.372 for the final model, a substantial proportion of outcome variation remains unexplained. The nomogram should therefore be regarded as an exploratory risk stratification tool pending external validation, rather than a definitive clinical decision instrument (18). Fourth, surgical and perioperative protocols evolved during the study period (2015–2025). We conducted a sensitivity analysis comparing outcomes between the early (2015–2019) and late (2020–2025) periods, which showed no significant difference in wound healing rates (*P* = 0.387), suggesting that protocol evolution did not substantially bias our findings. However, future prospective studies should incorporate standardized protocol documentation (19, 20).

This study has several limitations. First, it was conducted at a single center with a retrospective design, which may introduce selection bias and limits generalizability. Second, the validation performed in this study—comprising bootstrap resampling and a random hold-out split within the same institution—constitutes internal validation only. Although these procedures reduce overfitting and provide a preliminary estimate of model performance, they do not establish transportability to other institutions, surgical teams, ethnic groups, or perioperative protocols. External validation in geographically and demographically diverse multicenter cohorts is a prerequisite before the model can be considered ready for routine clinical use. Third, the relatively modest pseudo-R^2^ of 0.372 for the final model indicates that a substantial proportion of outcome variation remains unexplained by the five included predictors, which should be acknowledged as a limitation of the model's explanatory capacity. Fourth, some variables such as diabetic peripheral neuropathy were assessed clinically without fully standardized quantitative tools in all cases, which may affect measurement consistency; we have now applied standardized diagnostic criteria as described in the Methods and conducted a sensitivity analysis to address this concern. Fifth, several important surgical and perioperative variables were not included in the prediction model. It should be noted, however, that these variables exhibited minimal variability in our cohort due to standardized institutional protocols: all procedures were performed by the same surgical team using an identical lateral L-shaped approach with plate fixation, without drains; soft-tissue swelling was routinely confirmed resolved before incision; prophylactic antibiotics followed a uniform preoperative protocol; and all patients received insulin-based glycemic control. Because these factors were essentially constant across the study population, they would not have added predictive discrimination. This homogeneity limits generalizability to centers where practice patterns vary. Finally, the proposed risk-based management guidance in this manuscript is exploratory and based on the internally validated predictors; it should not be regarded as evidence-based clinical guidelines until prospective impact studies confirm that model-guided care improves patient outcomes.

The findings of this study provide clinicians with a preliminary risk estimation framework to identify type 2 diabetic patients at elevated risk for impaired wound healing after calcaneal fracture surgery. The internally validated nomogram is based on five predictors derived directly from the model (HbA1c, insulin use, diabetic peripheral neuropathy, CRP, and platelet count). It should be noted that this tool has not yet undergone external validation, and the risk-stratification categories and management recommendations described below are expert-opinion-based exploratory guidance rather than a validated clinical algorithm. Specific risk thresholds (e.g., low risk <20%, moderate risk 20%–40%, high risk ≥40%) were selected to illustrate clinical scenarios and require external validation. In the internal validation cohort, these thresholds classified 58.9% (53/90), 21.1% (19/90), and 20.0% (18/90) of patients as low, moderate, and high risk, with observed event rates of 5.7%, 26.3%, and 77.8%, respectively. The specific management strategies listed—including HbA1c targets, CRP-based surgical delay, and offloading protocols—were not directly tested or validated by this study and reflect current clinical consensus rather than model-derived evidence. Clinicians are encouraged to supplement these with individualized clinical judgment regarding surgical timing, approach, and perioperative management.

## Conclusion

In the present study, a nomogram for predicting impaired wound healing after calcaneal fracture surgery in T2DM patients was developed and internally validated using bootstrap resampling and a hold-out validation set. The model demonstrated acceptable discriminative ability (AUC 0.886 in training; 0.891 in validation), satisfactory calibration, and favorable net benefit over default strategies across threshold probabilities from 0.01 to 0.95. The five model predictors—HbA1c, insulin use, diabetic peripheral neuropathy, CRP, and platelet count—are all routinely available preoperatively and reflect the metabolic and inflammatory pathophysiology underlying wound vulnerability in this population. This nomogram represents a promising exploratory tool for preoperative risk stratification; however, it is limited by its single-center retrospective design and the absence of external validation. Accordingly, the model should be interpreted cautiously, and broad clinical adoption is premature until prospective external validation in independent, multicenter cohorts has been completed.

## Data Availability

The raw data supporting the conclusions of this article will be made available by the authors, without undue reservation.
