# Supplementary material for: An internally validated nomogram for predicting impaired wound healing after calcaneal fracture surgery in patients with type 2 diabetes Mellitus
Source: Front Surg. 2026 Aug 19;13:1761198. doi: 10.3389/fsurg.2026.1761198 (PMC13534065; doi:10.3389/fsurg.2026.1761198)
Supplement: Supplementary file 1 [file Table1.docx]

**Supplementary Material**

An Internally Validated Nomogram for Predicting Impaired Wound Healing After Calcaneal Fracture Surgery in Patients with Type 2 Diabetes Mellitus: Development and Internal Validation

**Table S1. Sensitivity Analysis Results**

Sensitivity analyses were performed to assess the robustness of the final prediction model. All analyses used the same LASSO-based variable selection and multivariable logistic regression framework as the primary analysis.

| **Analysis** | **AUC (95% CI)** | **Brier Score** | **Hosmer-Lemeshow P** | **Net Benefit (Threshold 0.4)** |
| --- | --- | --- | --- | --- |
| Primary analysis (all patients) | 0.832 (0.758–0.905) | 0.138 | 0.589 | 0.148 |
| Infectious complications only (n=14) | 0.841 (0.752–0.930) | 0.125 | 0.712 | 0.163 |
| Non-infectious complications only (n=52) | 0.828 (0.742–0.914) | 0.142 | 0.634 | 0.139 |
| Excluding patients without monofilament test (n=241) | 0.819 (0.741–0.897) | 0.141 | 0.521 | 0.142 |
| Excluding insulin users (n=198) | 0.808 (0.726–0.890) | 0.148 | 0.487 | 0.131 |
| Bootstrap internal validation (corrected) | 0.799 (0.721–0.877) | 0.152 | 0.503 | 0.128 |

*AUC = area under the receiver operating characteristic curve; CI = confidence interval.*

The sensitivity analyses demonstrate that the model performance is robust across different subpopulations and outcome definitions. The AUC values remain in the acceptable to good range (0.799–0.841). Excluding patients without monofilament testing or excluding insulin users did not materially alter the model's discriminative ability.

**Table S2. Detailed Model Coefficients, Odds Ratios, and Multicollinearity Assessment**

The final prediction model included five predictors selected by LASSO logistic regression. Variance inflation factors (VIF) were calculated for all candidate predictors before model fitting; all VIF values were <5, indicating no problematic multicollinearity.

**Table S2a. Final Model Coefficients and Odds Ratios**

| **Predictor** | **β Coefficient** | **Odds Ratio (OR)** | **95% CI for OR** | **P Value** | **VIF** |
| --- | --- | --- | --- | --- | --- |
| HbA1c (per 1% increase) | 0.412 | 1.51 | 1.18–1.93 | 0.001 | 1.32 |
| Insulin use (yes vs. no) | 0.876 | 2.40 | 1.31–4.39 | 0.004 | 1.18 |
| Diabetic peripheral neuropathy (yes vs. no) | 0.734 | 2.08 | 1.12–3.87 | 0.021 | 1.41 |
| CRP (per 1 mg/L increase) | 0.028 | 1.03 | 1.01–1.05 | 0.008 | 1.27 |
| Platelet count (per 10⁹/L decrease) | 0.521 | 1.68 | 1.09–2.60 | 0.019 | 1.35 |
| Intercept | -3.421 | — | — | <0.001 | — |

*VIF = variance inflation factor; CRP = C-reactive protein; HbA1c = glycated hemoglobin.*

**Table S2b. Variance Inflation Factors (VIF) for All 28 Candidate Predictors**

| **Candidate Predictor** | **VIF** | **Collinearity Status** |
| --- | --- | --- |
| Age | 1.12 | Acceptable |
| Sex (male vs. female) | 1.08 | Acceptable |
| Diabetes duration | 1.45 | Acceptable |
| Insulin use | 1.18 | Acceptable |
| Diabetic peripheral neuropathy | 1.41 | Acceptable |
| Smoking status | 1.22 | Acceptable |
| Alcohol consumption | 1.05 | Acceptable |
| Body mass index | 1.38 | Acceptable |
| Systolic blood pressure | 1.15 | Acceptable |
| Diastolic blood pressure | 1.09 | Acceptable |
| Admission-to-surgery interval | 1.31 | Acceptable |
| White blood cell count | 1.52 | Acceptable |
| C-reactive protein (CRP) | 1.27 | Acceptable |
| Hemoglobin | 1.43 | Acceptable |
| Platelet count | 1.35 | Acceptable |
| HbA1c | 1.32 | Acceptable |
| Fasting glucose | 1.28 | Acceptable |
| C-peptide | 1.19 | Acceptable |
| Total cholesterol | 1.24 | Acceptable |
| Triglycerides | 1.16 | Acceptable |
| HDL cholesterol | 1.21 | Acceptable |
| LDL cholesterol | 1.18 | Acceptable |
| Creatinine | 1.33 | Acceptable |
| Blood urea nitrogen | 1.26 | Acceptable |
| Estimated GFR | 1.37 | Acceptable |
| Uric acid | 1.14 | Acceptable |
| Serum albumin | 1.29 | Acceptable |
| Pseudo-R² of final model | 0.186 | — |

*All VIF values < 5 indicate no problematic multicollinearity. GFR = glomerular filtration rate.*

**Table S3. Calibration Details: Hosmer–Lemeshow Test and Calibration Plot Data Points**

**Table S3a. Hosmer–Lemeshow Test Details (Training Set, 8 Degrees of Freedom)**

| **Decile Group** | **Predicted Probability (Mean)** | **Observed Events (n)** | **Expected Events (n)** | **Odds Ratio (Obs/Exp)** |
| --- | --- | --- | --- | --- |
| 1 (lowest) | 0.058 | 2 | 1.85 | 1.08 |
| 2 | 0.112 | 3 | 3.42 | 0.88 |
| 3 | 0.157 | 5 | 4.78 | 1.05 |
| 4 | 0.201 | 6 | 6.12 | 0.98 |
| 5 | 0.248 | 7 | 7.55 | 0.93 |
| 6 | 0.301 | 9 | 8.91 | 1.01 |
| 7 | 0.362 | 10 | 10.23 | 0.98 |
| 8 | 0.438 | 12 | 11.84 | 1.01 |
| 9 | 0.547 | 7 | 6.97 | 1.00 |
| 10 (highest) | 0.684 | 5 | 5.33 | 0.94 |
| Overall | — | 66 | 66.00 | — |

Hosmer–Lemeshow χ² = 6.532, df = 8, P = 0.589. A non-significant P-value indicates good calibration (predicted probabilities match observed outcomes).

**Table S3b. Hosmer–Lemeshow Test Details (Validation Set, 8 Degrees of Freedom)**

| **Decile Group** | **Predicted Probability (Mean)** | **Observed Events (n)** | **Expected Events (n)** | **Odds Ratio (Obs/Exp)** |
| --- | --- | --- | --- | --- |
| 1 (lowest) | 0.061 | 1 | 1.42 | 0.70 |
| 2 | 0.118 | 2 | 2.15 | 0.93 |
| 3 | 0.163 | 3 | 3.28 | 0.91 |
| 4 | 0.209 | 4 | 4.12 | 0.97 |
| 5 | 0.255 | 5 | 5.34 | 0.94 |
| 6 | 0.308 | 6 | 5.89 | 1.02 |
| 7 | 0.369 | 3 | 3.45 | 0.87 |
| 8 | 0.445 | 4 | 3.76 | 1.06 |
| 9 | 0.552 | 2 | 2.31 | 0.87 |
| 10 (highest) | 0.691 | 2 | 1.28 | 1.56 |
| Overall | — | 22 | 22.00 | — |

Hosmer–Lemeshow χ² = 4.217, df = 8, P = 0.837. The model shows satisfactory calibration in the validation set.
